# Consistent Condom Use by Female Sex Workers in Kolkata, India: Testing Theories of Economic Insecurity, Behavior Change, Life Course Vulnerability and Empowerment

**DOI:** 10.1007/s10461-016-1412-z

**Published:** 2016-05-11

**Authors:** Anne E. Fehrenbacher, Debasish Chowdhury, Toorjo Ghose, Dallas Swendeman

**Affiliations:** 1 Department of Community Health Sciences, Fielding School of Public Health, University of California, Los Angeles, 650 Charles E Young Drive South, Los Angeles, CA 90095 USA; 2 Public Health Foundation of India (PHFI), Sector-44, Plot No-47, Gurgaon, 122003 India; 3 School of Social Policy & Practice, University of Pennsylvania, 3701 Locust Walk, Caster D17, Philadelphia, 19104 USA; 4 Center for HIV Identification, Prevention and Treatment Services (CHIPTS), Department of Psychiatry and Biobehavioral Science, David Geffen School of Medicine, University of California, Los Angeles, 10920 Wilshire Blvd., Suite 350, Los Angeles, CA 90024 USA; 5 Department of Epidemiology, Fielding School of Public Health, 10920 Wilshire Blvd., Suite 350, Los Angeles, 90024 USA

**Keywords:** Community mobilization, Structural interventions, Sex work, Empowerment, Economic insecurity

## Abstract

Consistent condom use (CCU) is the primary HIV/STI prevention option available to sex workers globally but may be undermined by economic insecurity, life-course vulnerabilities, behavioral factors, disempowerment, or lack of effective interventions. This study examines predictors of CCU in a random household survey of brothel-based female sex workers (n = 200) in two neighborhoods served by Durbar (the Sonagachi Project) in Kolkata, India. Multivariate logistic regression analyses indicated that CCU was significantly associated with perceived HIV risk, community mobilization participation, working more days in sex work, and higher proportion of occasional clients to regular clients. Exploratory analyses stratifying by economic insecurity indicators (i.e., debt, savings, income, housing security) indicate that perceived HIV risk and community mobilization were only associated with CCU for economically secure FSW. Interventions with FSW must prioritize economic security and access to social protections as economic insecurity may undermine the efficacy of more direct condom use intervention strategies.

## Resumen

El uso consistente del condón (CCU) es la principal opción de prevención de VIH/SIDA a disposición de los trabajadoras sexuales a nivel mundial, pero puede verse afectada por la inseguridad económica, las vulnerabilidades de ciclo de vida, factores de comportamiento, pérdida de poder, o la falta de eficacia de las intervenciones. Este estudio examina los predictores de CCU en una encuesta de hogares al azar de las trabajadoras sexuales en prostíbulos (n = 200) en dos barrios atendidos por Durbar (el Proyecto Sonagachi) en Calcuta, India. Los análisis de regresión logística multivariante indicó que CCU se asoció significativamente con el riesgo de VIH percibida, la participación movilización de la comunidad, trabajar más días en el trabajo sexual, y una mayor proporción de clientes ocasionales a los clientes regulares. Los análisis exploratorios estratificar por los indicadores de inseguridad económica (es decir, la deuda, el ahorro, ingresos, seguridad de la vivienda) indican que la percepción de riesgo de VIH y movilización de la comunidad se asocian únicamente con CCU para la seguridad económica de FSW. Las intervenciones con FSW deben dar prioridad a la seguridad económica y el acceso a las protecciones sociales como la inseguridad económica puede poner en peligro la eficacia de las estrategias más el uso de condones de intervención directa.

## Introduction

The first reported cases of HIV in India were diagnosed among female sex workers (FSW) in Chennai, Tamil Nadu in 1986 [1]. Since that time, FSW have been regarded as one of the most high-risk populations behind the rising tide of HIV/AIDS in South Asia. India’s epidemic has followed a “Type 4” pattern in which new infections occur first among vulnerable groups, such as sex workers and injection drug users, and then spread to bridge populations, such as clients of sex workers, who transmit the virus to the general population [2]. Today, India has the third largest population of HIV-infected persons in the world with approximately 2.1 million cases, despite a low overall prevalence of 0.3 % [3].

Incidence and prevalence of HIV among FSW varies greatly by locale in India [2]. Concentrated epidemics among FSW have been documented with greater than 50 % prevalence in Bombay (Mumbai) and Delhi since the late 1990s [4]. The variation in disease burden among FSW across India has been attributed to the uptake of empowerment-based interventions in certain regions, such as West Bengal [5]. Empowerment-based interventions incorporate community mobilization (e.g., door-to-door educational outreach, promotion of civic engagement, organizing for political actions), advocacy (e.g., pressure on local authorities to prevent institutional discrimination, local and national policy changes to benefit FSWs and their families, etc.), and economic strategies (e.g., microfinance, cooperative banking, job training, etc.) to supplement traditional intervention activities such as provision of condoms, and testing and treatment for sexually transmitted infections (STI) and HIV.

The Durbar intervention in Kolkata, West Bengal (a.k.a., the Sonagachi Project) is the longest running empowerment program for sex workers in India [6]. Durbar is considered a national and global model intervention with sex workers [7], including serving as a model for the Gates Foundation’s Project Avahan scale-up of HIV prevention for high-risk groups in India [8]. Although there is a relatively large body of research examining Durbar’s intervention processes [9–12], only a few studies examine the impacts of multi-component empowerment interventions with sex workers generally [5] and with Durbar specifically [13, 14]. Furthermore, the relative impacts of multiple intervention strategies embedded within empowerment interventions, and the theoretical mechanisms linking these strategies to HIV/STI risk behaviors, remain poorly conceptualized [5].

Because there is no effective vaccine or cure for HIV, and pre-exposure prophylaxis (PrEP) is not widely accessible to the population in this study [15, 16], at present, CCU remains the most effective method for preventing transmission of HIV, and the only method for preventing STIs, among FSW in India [17].

The objective of the current paper is to test multiple theory-based hypotheses for predicting consistent condom use (CCU) by FSW with their clients in two Kolkata neighborhoods served by Durbar for the last 20 years. The research questions for this analysis are: (1) What factors are associated with CCU among FSW in the presence of the Durbar intervention? (2) Do correlates of CCU vary between economically secure and economically insecure FSW?

## Theoretical Approaches to Predicting Consistent Condom Use

In this paper, we test correlates of CCU among FSW organized by four theoretical domains: life-course theories [18–21]; economic security theories [22–25]; health behavior change theories [26–28]; and empowerment theories that elaborate the interplay of structure and agency [29–32]. In addition, we examined multiple variables reflecting exposure to and participation in Durbar’s intervention activities. Prior research with FSW identified the above domains as important factors influencing CCU, specifically, such as risk perceptions, personal and community empowerment attitudes, vulnerabilities associated with life course circumstances (e.g., entry into sex work via trafficking), instrumental services (e.g., provision of condoms, intervention exposure and participation), and economic empowerment (e.g., financial independence) [22, 25, 33–35].

Life-course theories suggest that adverse life events and cumulative disadvantage [18–21] impact marginalization and disempowerment, which could undermine intervention effects. Life course factors are reflected in demographic background variables such as age of entry into sex work, time in sex work, current age, education, coerced or forced entry into sex work (i.e., trafficking into sex work), and exposure to violence in the workplace. Durbar targets these factors primarily through anti-trafficking self-regulatory boards [36, 37], advocacy and intervention by sex worker community associations, and education programs with husbands and partners [38]. We propose hypotheses based on the assumption that stressful or traumatic life events have long-term adverse effects that may not fully resolve even when stressful or coercive conditions are no longer present, impairing perceived control and self-efficacy, and leading to less consistent condom use [39–41]. We hypothesize that trafficking into sex work and recent violent experiences may decrease CCU by limiting agency and power. We also hypothesize that current age has a curvilinear association with CCU, since young FSW may have less power to negotiate with clients, but older FSW may have fewer clients and thus may be more economically insecure to negotiate effectively [42, 43].

Economic theories elaborating expected utility, heuristics, and loss aversion in decision-making [23–25, 35] suggest that perceived economic security and insecurity indicated by income, debt status, or savings may influence condom negotiation and behaviors [22, 33]. Prior research indicates that FSW in Kolkata who insist on using condoms with all clients are estimated to suffer a wage penalty of 66–79 % compared to those who do not consistently use condoms [23]. Durbar primarily targets economic vulnerabilities through their robust USHA Multipurpose Cooperative Bank with active savings outreach and mentored lending to mitigate economic shocks and debt bondage. We hypothesize that variables related to economic security are positively associated with CCU. For example, we expect price per client, total number of clients, weekly income, and having savings to be positively associated with CCU, while being in debt is posited to be negatively associated with CCU [22, 23, 25, 44, 45].

Health behavior change theories based on value-expectancies balancing perceived risk and reward (e.g., Health Belief Model, Theory of Reasoned Action) are widely used to design condom use interventions in the fields of public health and psychology [26–28]. These theories suggest that perceived risk (i.e., susceptibility) for HIV/STI and perceived barriers and rewards for using condoms impact CCU. Durbar targets value-expectancies primarily through peer community health workers (CHW) conducting daily workplace condom social marketing and bi-monthly (twice per month) peer education in routine home and workplace visits. We hypothesize that CCU is positively associated with perceived risk for HIV, positive condom attitudes, and knowing one’s HIV status. We also hypothesize that higher numbers of clients and days of sex work per week potentially reflect both higher perceived risk for HIV/STI but also heightened economic security [46–48]. Sex with regular clients may also be associated with less CCU compared to sex with occasional clients due to a decrease in perceived risk based on familiarity or intimacy with regular clients [49, 50].

Empowerment theories related to autonomy or agency [29], critical consciousness around sources of marginalization [30], and the consequent attention to the interplay between structure and agency [31, 32], suggest that empowerment attitudes (e.g., collective identity, self-efficacy, mutual support, critical consciousness) and negotiation skills influence CCU by FSW [51]. Durbar targets these empowerment domains primarily by community mobilization activities such as street rallies and demonstrations, conferences, advocacy with power brokers, voter mobilizations, and political action. We hypothesize that empowerment-related variables including perceived and objective autonomy with clients (i.e., expected and experienced negotiation capacities) and empowerment attitudes regarding the legitimacy of sex work as work are positively associated with CCU. Finally, we also examine empowerment indicators related to participation in Durbar intervention activities (e.g., participation in mobilization activities, membership status, leadership roles, etc.) as hypothesized correlates of CCU, in addition to variables reflecting more general exposure to and participation in Durbar’s instrumental intervention activities. Previous studies to test the empowerment and life course explanations have found mixed results [11, 52–54].

Most broadly, we hypothesize that economic factors, both perceived and objective, will be the strongest predictors of condom use and CCU based on the growing body of literature on compensating wage differentials for sex workers to accept more money to perform unprotected sex [22, 25, 55]. Therefore, we examine the influence of economic insecurity on CCU via models stratified on savings, debt, income, and housing security.

**Fig. 1:**
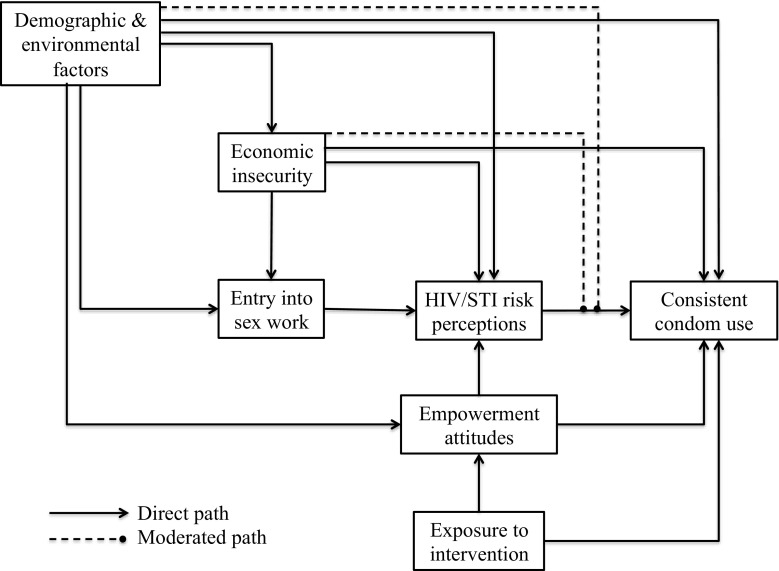
Integrated theoretical model of life course vulnerabilities, economic insecurity, risk perceptions, and empowerment attitudes influencing consistent condom use among female sex workers in India

An integrated theoretical model demonstrating how the constructs in each of the theories are related to one another is displayed in Fig. 1. Life course vulnerabilities are depicted in the figure by the demographic and environmental factors, which lead to different reasons for entry into sex work [43]. These background characteristics also influence all other variables in the model directly, indirectly through mediated processes, and as potential modifying factors altering the strength of the relationship between risk perceptions and consistent condom use behaviors. Economic insecurity is conceptualized as an antecedent, mediator, and moderator. Finally, exposure to the Durbar intervention influences empowerment attitudes, which in turn, influence consistent condom use directly and via the mediator of HIV/STI risk perceptions.

Figure 1 is designed to integrate the constructs and relationships hypothesized across all four theoretical domains discussed above, but not all relationships in the model are tested in this analysis. For the purpose of this analysis, the moderated path between HIV/STI risk perceptions and consistent condom use modified by economic insecurity is of particular interest for the hypotheses about group differences based on economic status and perceptions. We test the main effects of variables related to life course vulnerabilities, health behavior change, economic insecurity, and empowerment attitudes on CCU in addition to testing interaction effects based on client composition and economic insecurity indicators.

## Methods

### Data

The institutional review boards of Durbar and the University of California, Los Angeles reviewed and approved the study protocol. All participants provided informed consent. A cross-sectional demographic survey was administered in 2007 to 2008 through face-to-face interviews with 200 sex workers in randomly-selected households in two large red light areas in Kolkata (Sonagachi and Bowbazar) served by Durbar since 1992. Sex worker households (i.e., brothel rooms where sex workers work and typically live) were enumerated in a multi-stage process beginning with identifying and enumerating brothel buildings, enumerating rooms occupied by sex workers within brothels, then enumerating sex workers within rooms if more than one occupied the room. Participants were then randomly selected from the enumeration list. The enumeration, recruitment, and interviewing were conducted by the Durbar evaluation team comprised of bachelor and masters degree level staff accompanied by FSW peer staff. Fourteen participants voluntarily withdrew from the study before completing the survey due to time limitations. Three participants were withdrawn by interviewers due to interruptions by madams or male partners, and two were withdrawn due to inconsistent responses. Nineteen additional participants were randomly selected to replace the withdrawals to meet the target sample size (n = 200).

### Measures

The survey questionnaire was based on an instrument developed for evaluation of the Gates Foundation’s Project Avahan [56]. The objectives of the study were to measure demographic and socio-economic characteristics of FSW; knowledge, attitudes, and behaviors related to HIV/STIs; empowerment constructs; and exposure to and participation in Durbar intervention activities.

### Dependent Variables

The three dependent variables in this analysis are dichotomous outcomes for condom use with last occasional client, condom use with last regular client, and consistent condom use (CCU) with both last occasional and last regular client based on two questions: *“The last time you had sex with your last [regular, occasional] client, did you use a condom?”* (yes/no).

### Independent Variables

The primary demographic background variables used as independent variables to test *life course hypotheses* were current age in years, marital status (currently married or not), cohabitation (living with a spouse or sexual partner or not), and reason for entry into sex work. Reason for entry into sex work was operationalized using a set of seven yes/no questions regarding “the reasons you entered sex work,” that included: needed money, to gain independence, to get out of family violence, to get out of hard work, was advised by someone, was a traditional sex worker, or “was lured, cheated, or forced” into sex work. Other variables examined in this domain included exposure to violence in the last six months (yes/no), location of work (Sonagachi or Bowbazar), age of entry into sex work, years in sex work, education, and number of dependents.

The primary independent variables to test the behavior change theory *value*-*expectancy hypotheses* were perceived risk for HIV (yes/no), condom knowledge (low vs. high from a multi-item scale), and knowledge of HIV status (i.e., was tested for HIV within last six months and know the results vs. was not tested or did not find out results). Other variables examined in this domain include HIV knowledge (low vs. high from a multi-item scale), self-reported condom use with all clients in the last 30 days (yes/no), experienced STI symptoms in the last six months (yes/no), received treatment for STIs if needed (yes/no), and know someone personally who is living with HIV/AIDS (yes/no).

The primary independent variables for the *economic security hypotheses* are price for last client, weekly income, debt status, savings behavior, financial independence (“do not depend on others for money”), and housing security. Price for last client and weekly income are continuous variables measured in rupees that are log-transformed to approximate a normal distribution. Debt and savings are dichotomous (yes/no) variables from two questions assessing the last 12-month period. Other variables examined in this domain include has a bank account (yes/no) and has borrowed money in the last 12 months (yes/no).

Several variables could reflect either or both *value*-*expectancy (behavior change)* and *economic security* hypotheses; total number of clients, client composition, and number of days of sex work, all assessed for the last seven days. Client composition is constructed as two variables: one is a ratio of the number of regular clients in the last week divided by the number of occasional clients in the last week, and the second is an interaction term for the product of the number of regular clients in the last week by the number of occasional clients in the last week.

The primary independent variables for the *empowerment hypotheses* are five measures of empowerment attitudes, condom negotiations, and refusals of clients. Five multi-item scales were constructed from a confirmatory factor analysis of 26 empowerment attitude questions addressing: perceived collective identity with other sex workers, perceived autonomy with clients, perceived legitimacy of sex work as work, perceived political self-efficacy, and perceived institutional discrimination against sex workers. In addition, FSW were asked if they convinced a client to use a condom and refused a client for any reason in the last 30 days (yes/no). Other variables examined in this domain include “was offered more money for sex without a condom” within the last five years, and “accepted more money for sex without a condom” within the last five years.

The primary variables examined for *intervention participation hypotheses* include membership in Durbar, participation in a door-to-door community mobilization “education” campaign (as an unpaid member of Durbar to network and increase membership), served as a peer educator (paid part-time position), participation in workshops to instruct other sex workers about HIV/AIDS, participation in conferences, participation in political rallies, all dichotomous variables (yes/no). Intervention exposure variables include number of visits from DMSC in the last 30 days and having received pamphlets about HIV/AIDS from DMSC within the last 6 months.

### Statistical Analysis

All analyses were performed in Stata 12.1. Univariate descriptive statistics and bivariate associations with each dependent variable were computed for each covariate. Skewed variables were either log-transformed or the range was top-coded to account for outliers that were altering the distribution. Multivariate logistic regressions were conducted to identify significant correlates of condom use with last occasional client (outcome one), last regular client (outcome two), and CCU (outcome three). Multivariate models for each outcome were built by selecting all covariates associated with each outcome in the bivariate regressions below the 10 % significance Covariates were grouped by each theoretical domain and added successively to the multivariate models. Significant predictors in the final multivariate models were determined by a significance level of 5 % (*p* value of 0.05 or less).

Post-test procedures were used to assess model calibration and predictive accuracy. The fit of each of the final models was confirmed using the Hosmer–Lemeshow Goodness-of-Fit Chi squared test, the Bayesian Information Criterion (BIC), and the Akaike Information Criterion (AIC). The predictive accuracy of each model was assessed using the pseudo-R^2^ value and the area under the receiver operating characteristic (ROC) curve.

Interaction terms were added and goodness-of-fit statistics recomputed. Interactions were graphed using the margins and marginsplot commands in Stata 12.1. Due to small sample sizes in some cells of categorical variables that made it difficult to detect significant interactions with most variables, we reran the final CCU models stratifying on indicators of economic insecurity based on income, debt status, savings, and housing security. Parameter tests were performed to test the fit of the stratified models as described above.

## Results

### Characteristics of Sample

#### Background Characteristics

Table 1 shows descriptive statistics of the sample. FSW in the sample were well-established in the sex work communities in which they worked, with the majority having been in the sex work profession for nearly a decade. Almost two-thirds were recruited in the larger Sonagachi neighborhood and 36 % in Bowbazar. Ages ranged from 18 to 55 years old (mean = 30 years). The mean age at entry into sex work was 21 years, and 22 % entered under age 18. About one-quarter (22 %) worked in sex work for one year or less and 31 % for more than 10 years (mean = 8 years). More than three-quarters reported entering sex work due to financial need, with 21 % to gain independence, 17 % being trafficked or coerced, and 14 % entering to evade family violence (note that reasons for entering were not mutually exclusive).

**Table 1:** Demographic, financial, participation and empowerment characteristics of brothel-based female sex workers in Kolkata, India (n = 200)

Variable	%	n	Mean	SD
Demographic characteristics				
Age (range 18–55 years)			29.5	6.9
Years of schooling (0–12 years)			2.3	3.3
No formal education	60.5	121		
Class 1–6	24.5	49		
Class 7–12	15.0	30		
Age of entry into sex work (12–40 years)			21.2	5.0
Less than 18 years old	20.5	41		
18 years or older	79.5	159		
Time in sex work (0–39 years)			8.3	8.3
Experienced physical violence past 6 months	20.0	40		
Reason for entering sex work (not mutually exclusive)				
In need of money	78.0	156		
Advised by friend or relative	11.5	23		
Was a traditional sex worker	2.5	5		
Lured, cheated, or forced into sex work	16.5	33		
To get independence	20.5	41		
To get out of family violence	14.0	28		
To get out of hard work	6.0	12		
Economic security				
Number of days of sex work past week			4.5	2.1
Number of clients past week (range 0–45)			9.8	8.2
Regular clients past week (0–14 clients)			2.9	3.1
Occasional clients past week (0–41 clients)			7.0	7.2
Average weekly income from sex work (50–10,000 INR)			1530.9	1543.1
50–500 INR	24.0	48		
501–1000 INR	32.1	64		
1001–2000 INR	25.0	50		
2001–10,000 INR	18.9	38		
Number of dependents (0–11)			3.1	2.1
No dependents	10.5	21		
At least one dependent	89.5	179		
Financially independent	63.6	126		
Secure housing	69.0	138		
Has bank account	56.5	105		
Has an USHA cooperative passbook	52.5	113		
Saved money past year	67.5	135		
Borrowed money past year	39.5	79		
Currently in debt	34.5	69		
Durbar intervention participation & exposure				
Member of Durbar	63.0	126		
Served as a peer educator at Durbar past 6 months	23.0	46		
Level of participation in Durbar (0–3, low to high)			1.2	1.1
Not a member	37.0	74		
Member but do not participate	21.0	42		
Member and participate infrequently	29.0	58		
Member and participate regularly	13.0	26		
Number of times visited by Durbar past month (0–5)			3.6	1.4
Received pamphlets about HIV/AIDS from Durbar past 6 months	30.5	61		
Participated in past 6 months:	73.0	146		
Workshops to instruct other sex workers	5.0	10		
Door-to-door educational (mobilization) campaigns	28.0	56		
Conferences	29.5	59		
Empowerment indicators				
Collective identity (full range 0–36, actual 6–36, low to high)			22.0	5.2
Perceived institutional fairness (range 0–12, unfair to fair)			8.3	2.8
Autonomy with clients (range 0–12, low to high)			10.1	2.6
I decide the number of clients to take (0–4, never to always)			3.4	1.1
I decide the type of sex to have with clients (0–4)			3.4	0.9
I decide how much to charge clients (0–4)			3.3	1.2
Political self-efficacy (full 0–16, actual 4–15, low to high)			9.1	1.8
Legitimacy of sex work (full 0–16, actual 3–15, low to high)			9.3	2.0
Proud of being a sex worker (full 0–4, never to always)			2.5	1.3
“Always” proud of being a sex worker (yes/no)	38.0	76		
Sexual risk knowledge, attitudes, behaviors				
Condom knowledge scale (0–3, low to high)			2.4	1.1
HIV knowledge scale (0–4, low to high)			4.5	0.7
Number of times had sex with regular clients past week (range 0–27)			3.5	4.0
Number of times had sex with occasional clients past week (range 0–35)			7.3	7.1
Always used a condom with all clients past month	88.5	177		
Convinced a client to use condom past month	87.5	175		
Refused a client for any reason past month	64.5	129		
Client offered more money for sex without condom past 5 years	31.5	63		
Accepted more money for sex without condom past 5 years	17.5	35		
Experienced physical violence past 6 months	20.0	40		
Experienced STI symptoms past 6 months	41.5	83		
Received STI treatment for STI symptoms past 6 months	40.0	80		
Perceived HIV risk	65.0	130		
Taken an HIV test past 6 months	49.0	98		
Knows results of last HIV test	48.0	96		
Used condom with last regular client (n = 199)	74.5	149		
Used condom with last occasional client (n = 199)	76.0	152		
Used condom with BOTH last regular AND last occasional client	62.0	124		

Most FSW had no formal education and only 15 % had more than six years of schooling. Half were married or cohabiting with a sexual partner. Almost all (90 %) had at least one dependent child or other relative that they were financially supporting (mean = 3 dependents). Experiences of physical violence in the last six months was reported by 20 % of participants.

#### Financial Characteristics

Participants worked, on average, five days in the last week as a sex worker, seeing approximately 10 clients in that week. On average, respondents had more than twice as many occasional clients as regular clients, although the ratio of occasional to regular clients and the range of total clients varied widely. About 33 % had only five clients in the last week and 25 % had 15 or more clients. Almost two-thirds (64 %) were financially independent and 70 % had secure housing. However, one third were in debt and 40 % had borrowed money in the last 12 months. Just over half had a bank account and two-thirds had saved money during the last 12 months.

#### Participation in Durbar Intervention

Nearly two-thirds of the sample identified as members of Durbar, but only 13 % participated in Durbar events regularly. Respondents reported visitation by a Durbar representative an average of 4 times in the last month. About one-third had received a pamphlet about HIV/AIDS from Durbar in the last six months. The most common form of engagement in Durbar activities over the prior six months was participation in political or street rallies, followed by conferences, and door-to-door mobilization and education campaigns.

#### HIV/STI Knowledge, Attitudes, and Behaviors

Respondents reported high levels of knowledge regarding HIV/AIDS. Two-thirds perceived themselves to be at risk for HIV/AIDS. Half had taken an HIV test in the last six months and all of those reported that the test was voluntary and received results. Eight out of ten FSW personally knew someone living with HIV/AIDS. Respondents also had high levels of knowledge regarding proper use of condoms for STIs. Four in ten sex workers reported experiencing at least one STI symptom in the last six months and of those who needed STI treatment all were able to receive treatment.

#### Empowerment Attitudes

Respondents reported a moderate level of collective identity with other sex workers and a high level of autonomy with clients, on average. Respondents felt that local institutions were fair to sex workers, except for police and reports of discrimination against sex workers. Over one-third (38 %) reported always being proud of being a sex worker. Most endorsed sex work as legitimate work. Most also reported a moderately high level of political self-efficacy to affect change to benefit sex workers.

#### Condom Use Behaviors

Almost all participants (90 %) reported always using condoms with all clients in the last month, however, only 76 % reported using a condom with their last occasional client, 75 % with their last regular client, and 62 % with both their last occasional client and last regular client (i.e., CCU). Almost all (89 %) reported convincing a client to use a condom when he did not want to within the last 30 days. Two-thirds refused a client within the last 30 days for any reason. Although a third of respondents had been offered more money for sex without a condom within the last five years, only 18 % reported accepting it.

#### Bivariate Logistic Regressions

Table 2 shows variables with bivariate associations with any of the dependent variables at p < 0.20 significance level. Thirty candidate variables were tested as predictors of condom use with last occasional client, last regular client, and CCU. Based on bivariate associations at p < 0.10, fourteen covariates were selected for the multivariate model on condom use with last occasional client, twelve covariates for last regular client, and twelve covariates for CCU.

**Table 2:** Bivariate odds ratios for predictors of condom use by partner type and consistent condom use (N = 200)

Variable	Last regular client	Last occasional client	Both clients (CCU)
Demographic characteristics			
Age at interview		0.943*	0.945**
Years in sex work		0.938***	0.944**
Age of entry into sex work		1.080*	1.054^+^
Reasons for entering sex work			
Financial need		1.928^+^	
Lured, cheated, or forced into sex work		0.347**	0.514^+^
Financial security indicator			
Lives with sexual partner	0.637^^^	0.416*	0.520*
# Days of sex work (past week)	1.627***	2.075***	1.921***
Total number of clients (past week)	1.154***	1.289***	1.190***
Number of regular clients (past week)	3.314***		1.221**
Number of occasional clients (past week)	1.072*	1.725***	1.180***
High income earner (>1500 INR/week)	1.669	1.971*	2.369**
Log of income		1.729**	1.500*
Has an USHA cooperative pass book	1.658^^^	0.655^^^	
Saved money past year	1.670^^^		1.658^^^
Borrowed money past year	0.658^^^		0.644^^^
Currently in debt	0.487*		0.641^^^
Participation in Durbar			
Member Durbar		0.420*	0.621^^^
Durbar participation (0–3, not member to regular)		0.728*	0.842^^^
Participated in door-to-door educational campaigns to build Durbar Network*	3.788**	4.343**	3.892***
Served as peer educator past 6 months*	2.229^+^		1.757^^^
Empowerment			
Legitimacy of sex work (−2 to 2, low to high, actual range = −1.6 to 1.2)	2.319**		1.706^+^
Refused a client for any reason past month		1.779^+^	
Sexual risk behaviors, knowledge and attitudes			
Condom knowledge scale (0–2, low to high)*	1.484**		1.247^+^
Report using condom with all clients past month	2.552*		1.926^^^
Accepted more money sex without condom (past 5 years)	0.506^+^		
Experienced physical violence past 6 months	1.788^^^	2.017^^^	2.481*
Perceived HIV risk	3.101***	1.635^^^	3.162***

Significance level ^ ^^ p < 0.20; ^ +^ p < 0.10; * p < 0.05; ** p < 0.01; *** p < 0.001

Odds Ratio estimates with p > 0.20 not presented

#### Multivariate Logistic Regressions

Table 3 shows results of multivariate logistic regression models for condom use with different client types and CCU. When controlling for other characteristics, condom use with last *occasional client* was most strongly associated with condom use with last regular client (OR = 6.02, 95 % CI 1.52–23.86, p = 0.011) and an interaction of the number of occasional clients by the number of regular clients in the last week (OR = 0.81, 95 % CI 0.71–0.94, p = 0.005). Figure 1 shows a graph of the interaction. The highest odds of condom use with last occasional client were observed among those with a high number of overall clients and a high proportion of occasional to regular clients. The odds of condom use with last occasional client were reduced with each additional regular client, but the effect was much steeper among those with a lower number of occasional clients. For example, the probability of condom use with last occasional client was more than 80 % for those with no regular clients but less than 40 % for those with six or more regular clients. Those with a high number of occasional clients did not exhibit a reduction in the probability of condom use with occasional clients until having over four regular clients, although the probability never dipped below 60 %.

**Table 3:** Odds Ratios and 95 % Confidence Intervals from Multivariate Logistic Regression Models of Condom Use with Last Regular Client, Last Occasional Client, and Both Client Types (N = 199)

Variable	Regular client		Occasional client		Both clients (CCU)	
	OR	95 % CI	OR	95 % CI	OR	95 % CI
Used a condom with last client (of alternate type)	16.02***	(3.28, 78.18)	6.02*	(1.52, 23.86)	N/A	N/A
Number of occasional clients × Number of regular clients in the last week			0.81**	(0.71, 0.94)	1.10**	(1.01, 1.18)
High condom knowledge	3.96**	(1.38, 11.36)				
Perceived HIV risk	2.92*	(1.11, 7.75)			3.11**	(1.31, 7.37)
Number of regular clients	15.06***	(4.90, 46.31)				
Participated in door-to-door education campaign					3.54**	(1.39, 8.99)
Served as a peer educator					2.65^+^	(0.98, 7.14)
Number of days of sex work in the last week					1.64***	(0.01, 0.11)
Pseudo-R^2^	0.51		0.56		0.42	

Significance level ^+^ p < 0.10, * p < 0.05, ** p < 0.01, *** p = 0.001

*CCU* Consistent condom use, *N/A* not applicable

Condom use with last *regular client* (also shown in Table 3) was most strongly associated with using a condom with last occasional client (OR = 16.02, 95 % CI 3.28–78.18, p < 0.001), number of regular clients in the last week (OR = 15.06, 95 % CI 4.90–46.31, p < 0.001), level of condom knowledge (OR: 3.96, 95 % CI 1.38–11.36, p = 0.01), and perceived risk for HIV (OR = 2.92, 95 % CI 1.11–7.75, p = 0.031). There was no significant interaction observed between the number of occasional clients and the number of regular clients on condom use with regular clients.

**Fig. 2:**
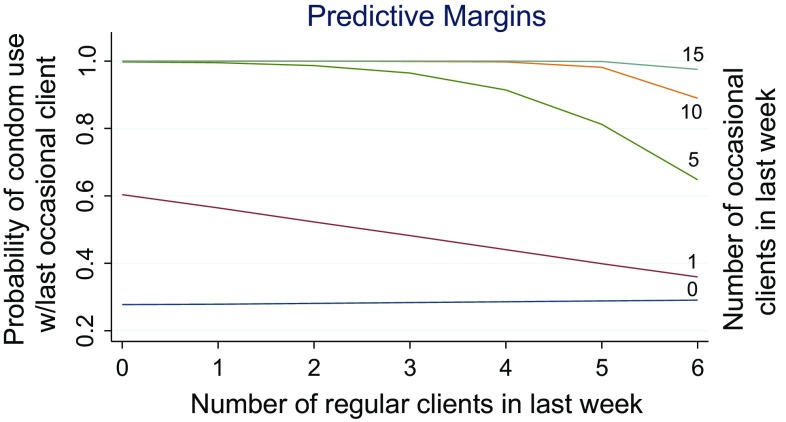
Graph of interaction of the number of occasional clients by the number of regular clients in the last week on predicted probabilities of condom use with last occasional client

*Consistent condom use* (CCU) with both last occasional client and last regular client (see Table 3) was most strongly associated with participating in door-to-door mobilization campaigns (OR = 3.54, 95 % CI 1.39–8.99, p = 0.008), perceived risk for HIV (OR = 3.11, 95 % CI 1.31–7.37, p = 0.010), and number of days of sex work in the last week (OR = 1.64, 95 % CI 1.25–2.16, p < 0.0001). The association between consistent condom use and serving as a peer educator was marginally insignificant (OR = 2.65, 95 % CI 0.98–7.14, p = 0.054). The interaction of the number of occasional clients by the number of regular clients was also associated with CCU (OR 1.10, 95 % CI 1.03–1.18, p = 0.004). Figure 2 shows a graph of interaction. Among those with a very low or moderately low number of occasional clients, the odds of CCU did not significantly change with each additional regular client. By contrast, for those with a medium or high number of occasional clients, the odds of CCU increased with each additional regular client.

#### Stratified Multivariate Logistic Regressions

**Fig. 3:**
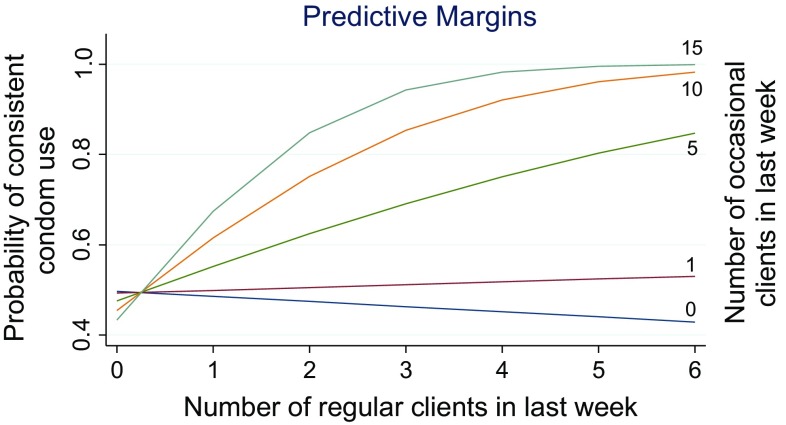
Graph of interaction of the number of occasional clients by the number of regular clients in the last week on predicted probabilities of consistent condom use (CCU) with both last regular and occasional clients

Table 4 shows results of analyses on predictors of CCU stratified by indicators of economic security, that is, debt, log of income, saving in the last 12 months, and housing security. In all of the stratified analyses, perceived risk for HIV and participation in a door-to-door mobilization campaign were only associated with increased odds of CCU among the economically secure strata. For those in the insecure strata on each economic indicator, only the number of days of sex work and the interaction of the number of occasional clients by the number of regular clients was associated with increased the odds of CCU (Fig. 3).

**Table 4:** Multivariate logistic regression models of consistent condom use (CCU) with both last occasional client and last regular client stratified by debt, savings, income, and housing status (N = 199)

Variable	Debt		Savings		Income		Housing	
	No	Yes	Saved	Did not save	High	Low	Secure	Insecure
Perceived risk for HIV	5.57** (1.61, 19.24)	2.23 (0.47, 10.55)	3.07* (1.08, 8.68)	7.31 (0.58, 91.62)	7.83** (2.09, 29.27)	1.32 (0.39, 4.52)	4.13* (1.38, 12.38)	1.16 (0.12, 11.28)
Participation in door-to-door mobilization	10.56*** (2.44, 45.66)	1.14 (0.23, 5.61)	4.79* (1.47, 15.68)	1.17 (0.12,11.88)	3.71^+^ (0.94, 14.64)	2.94 (0.73, 11.89)	9.31** (2.53, 34.24)	0.33 (0.01, 11.87)
Served as peer educator	2.24 (0.59, 8.54)	3.50 (0.59, 20.80)	2.65^+^ (0.86, 8.17)	4.36 (0.30, 63.66)	3.85 (0.65, 22.78)	3.14 (0.80, 12.37)	4.15* (1.14, 15.05)	1.78 (0.08, 36.76)
Number of days of sex work in the last week	1.66** (1.17, 2.36)	2.42* (1.21, 4.82)	1.50** (1.11, 2.03)	2.90* (1.16, 7.26)	1.62* (1.08, 2.41)	1.90** (1.22, 2.97)	1.79** (1.27, 2.54)	1.02 (0.49, 2.14)
Number of occasional clients × number of regular clients in the last week	1.06 (0.98,1.16)	1.24* (1.05, 1.47)	1.01 (0.99, 1.14)	1.44* (1.07, 1.94)	1.06 (0.98, 10.14)	1.20* (1.04, 1.38)	1.00 (0.94, 10.35)	1.48* (1.13, 1.96)
Pseudo-R^2^	0.46	0.45	0.35	0.68	0.48	0.37	0.44	0.68

Significance level ^+^ p  < 0.10,* p < 0.05, ** p < 0.01, *** p = 0.001

## Discussion

This study’s findings suggest that traditional public health interventions emphasizing education to alter knowledge and attitudes regarding condom use may not influence behaviors for those who are economically insecure. More specifically, the results suggest that there are four major determinants of consistent condom use among FSW, which vary in importance based on regular or occasional client type: condom use with other partner types, perceived economic security, regular and occasional client composition, and HIV/STI risk perceptions. Overall, this study’s findings support hypotheses linked to an integration of value expectancy theories of behavior change and economic decision-making. Although we tested associations between life course events, empowerment attitudes, and CCU, drawing claims about the effectiveness of empowerment-based intervention components was beyond the scope of this analysis as all participants and their communities had been exposed to Durbar’s empowerment activities for up to 15 years. Future research should evaluate the effectiveness of various community empowerment approaches and their effects on ameliorating life course vulnerabilities.

Stable income reflected in past week number of days of sex work and number of clients was a strong predictor of condom use across client types. Although days of work and number of clients are not direct measures of income, they may be considered proxies for economic stability that allow an FSW to feel confident in her income potential and demand for her services. The significant interactions of number of occasional clients by number of regular clients on odds of condom use with occasional clients and on CCU suggest the existence of an intimacy gradient in which sex workers are more likely to use condoms with occasional clients. Having more clients and, in particular, more occasional clients, was associated CCU. By contrast, having more regular clients was only a positive predictor of CCU when an FSW also reported a high number of occasional clients. Models stratified by indicators of economic insecurity demonstrate that risk perceptions influence condom use only for sex workers who are economically secure.

This study’s results suggest that it is crucial to evaluate the economic status of FSW when designing interventions to promote condom use. The majority of FSW in the sample had secure housing and were financially independent, although-one third reported being in debt and borrowing money in the past year. A majority also reported saving money during the last year despite only 40 % having a bank account. These seemingly counterintuitive findings suggest knowledge within the sex work community of the value of saving money to protect against economic shocks even without formal banking services. FSW in Kolkata have been wary of banking institutions due to exclusion and corruption from formal banks in the past [38]. One of Durbar’s earliest and most robust structural interventions was the establishment of their own banking cooperative, the USHA Multipurpose Cooperative, and more recently, advocacy work to support sex workers’ access to traditional banks using USHA documentation because birth certificates and other identity documents are not possessed by or easily accessible to many FSW in India.

The Durbar multipurpose banking cooperative has provided a venue for FSW to manage their earnings collectively and avoid the institutional discrimination and stigma they face in formal banking settings. Durbar’s sex-worker owned and operated banking model reduces disenfranchisement from financial and economic institutions and promotes the autonomy of sex workers by integrating strategies for reducing economic insecurity into existing community mobilization campaigns. For example, Durbar leaders teach FSW to negotiate their own prices and the conditions under which they will or will not perform certain services. Nonetheless, although economic security for FSW is an emerging priority for the Durbar intervention, barriers remain that limit the reach and effectiveness of existing programs. For example, declining incomes among aging FSW may not be sufficiently addressed through savings and lending programs. Alternative forms of revenue beyond sex work may be necessary for FSW who are not able to maintain a high number of clients and may feel inclined to have sex without condoms to accept additional money or to get or retain clients. Nonetheless, many FSW in the study population still live in poverty, so they may not insist on condom use even though they have the power and skill to make this demand, particularly if they have a low or inconsistent stream of clients from week to week. As a result, Durbar has set a priority to provide economic opportunities for older sex workers to transition into other paid roles within the organization or in micro-banking supported entrepreneurial activities in order to supplement their unstable sex work incomes.

FSW in the sample were well-acquainted with the Durbar intervention and its aims. More than half reported being members of the Durbar collective and participated in community mobilization actions such as door-to-door campaigns, political rallies, protests, and conferences. The preference for cooperative rather than individual forms of civic and political engagement among sex workers is not surprising given the strong emphasis of the Durbar intervention on community-building and collective identity formation [51]. This finding may also help to explain why high levels of perceived autonomy, collective identity, and endorsement of sex work as legitimate were not associated with condom use in this study. Given that most respondents had regular bi-weekly visits from Durbar outreach workers as well as other exposures, the high levels of knowledge regarding HIV/AIDS and condoms are not surprising.

Nearly all respondents reported convincing a client to use a condom when he did not want to and successfully refusing a client for any reason in the last month. Only a small number of sex workers reported ever accepting additional money for sex without a condom, although this measure may be biased since it is asked in terms of a client offer to pay more rather than a FSW setting her price higher for a risky behavior. It should also be noted that the majority of FSW did not report being offered more money for sex without a condom, which may suggest that the client population in this area does not have a strong preference for condomless sex, and/or that clients are aware of both condom use norms and support for refusal by power brokers (i.e., madams) and peers. Durbar advocates with brothel power-brokers and clients to make condom use the status quo while mobilizing collective identity among sex workers through street rallies, conferences, and peer education campaigns to frame condom use as issues of workers’ rights and occupational safety [51].

There are several limitations to this study that limit the causal inferences implied in the results. First, our analysis uses cross-sectional data, so we cannot untangle the temporal relationship between variables. Second, small sample sizes within certain subgroups (e.g., only 62 respondents reported not having secure housing) limited statistical power for interaction analyses. For example, although no significant interactions were observed based on any of the objective economic variables, such as income or debt status, the stratified models suggest that the relationships between risk perceptions and CCU may be conditional on perceived economic status. Stratified analysis results should be considered exploratory. Third, the survey instrument did not assess current HIV/STI infection beyond self-reported symptoms in the last six months, which misses asymptomatic infections. Finally, the dependent variables queried condom use with the last client only, which is a common method for reliably assessing condom use by FSW, but misses variability in condom use across multiple clients and encounters over time. By contrast, 88.5 % of the FSW responded that they “always” used condoms in response to the global question on condom use for the past month, which suggests that this type of measure reflects attitudes biased by normative expectations. Future research should query FSW regarding multiple clients and encounters to increase reliability around inferences on consistency of condom use.

## Conclusion

Durbar has been successful in reducing vulnerability to HIV infection related to sex work by addressing multiple economic, social, and health needs of FSW and their families through comprehensive, multi-level, instrumental services, community mobilization, empowerment strategies, and perhaps most importantly, economic security through access to safe and secure savings and lending through the USHA cooperative bank. The results of this study indicate that different intervention strategies and targets are important for different FSW population segments. The findings provide support for an integrative model of condom use behaviors among FSW that combines elements of value-expectancy theories of behavior change based on perceived risk and rewards along with theories of economic decision-making and insecurity. Interventions with FSW may be more effective if strategies are modified based on the economic status of different subgroups, such that economic stabilization and promotion of savings behaviors could be core strategies for FSW with precarious economic status, which would in turn support condom use and potentially other health behaviors such as treatment adherence. For economically secure FSW who do not use condoms consistently, emphases can be placed on sexual health education and changing risk perceptions. Supporting a basic level of economic security and social protections must be key priorities for future interventions to promote condom use in sex work communities.
